# Developing a third-degree burn model of rats using the Delphi method

**DOI:** 10.1038/s41598-022-18092-0

**Published:** 2022-09-02

**Authors:** Zhaoyan Chen, Ya Zeng, Fangyuan Tian

**Affiliations:** Department of Pharmacy, West China Hospital, Sichuan University, No. 37, Guoxue Lane, Chengdu, Sichuan 610041 China

**Keywords:** Diseases, Medical research

## Abstract

Animal experiments play an essential role in advances in the research and treatment of burns. Currently, researchers often rely on personal experience or the literature to complete the construction of animal disease models, which may lead to a lack of scientific rigor and a wide range of animal disease models with reference value. The purposes of this study were to establish a third-degree burn model of rats using the Delphi method to provide a reference. Two rounds of a Delphi expert consultation survey were conducted on experts (*n* = 13) in this study, and then the boundary value method was used to screen, modify and supplement the indicators. Next, the indicator weight was determined by dividing the boundary value, and finally, the index system of the rat model of third-degree burns was established. The statistical analysis results show that the positive coefficients of the two rounds of expert consultation are 100% and 88.67% respectively. The expert authority coefficient values were 0.73 and 0.67, respectively, and the expert coordination coefficient test was *P* < 0.001. According to the experts' suggestion, the third-degree burn model of rats with 8 first-degree indexes, 14 second-degree indexes and 46 third-degree indexes was finally established. According to the characteristics and quality requirements of animal models, this study constructs a rat model of third degree burns, which is expected to expressively improve the overall proficiency of burn research quality.

## Introduction

Burns are caused by heat (such as boiling fluids, hot metals, flames, steam, etc.), electricity, chemicals (such as strong acid, alkali, etc.), radiation and other causes of body damage, of which the majority of thermal burns. According to the statistics of the World Health Organization (WHO), there are up to 300,000 deaths caused by burns in the world every year^1^. Approximately 27 million people suffer burns in China each year, and approximately 130,000 people die of burns^2^. Burns have become a global public health issue. Burns also lead to a heavy financial burden. Studies have shown that the cost of treating 1% of total body surface area (TBSA) burns in the intensive care unit (ICU) is as much as 4600 euros^3^. In addition, burns also bring serious physiological and psychological harm to patients, and the social impact is extensive and huge.

Due to the limitation of medical ethics, the study of partial burns is not suitable for the human body. Therefore, animal experiments with burns are of the utmost importance. Animal experiments are an important bridge linking basic research and clinical research, and animal disease models are the cornerstone of scientific rigor in animal experiments. Animal model building research has greatly improved our understanding of disease mechanisms and the progress of treatment. However, in current studies on the construction of animal disease models, researchers often rely on personal experience or other researchers' literature to complete the construction of animal disease models, which may lead to a lack of scientific rigor and a wide range of animal disease models with reference value.

In the early stage of our study, we conducted a systematic review of the rat burn model construction experiment^4^ and found that in the process of model construction, there are various induction methods and great differences in the current rat burn model construction experiment, and the report on the model construction process is not detailed, which has a certain impact on the overall research quality. The reference value for follow-up research is limited. In addition, our previous study found that the number of third-degree burn model construction experiments in rats was greater. Therefore, this study selects the rat third-degree burn model construction method as the representative and, based on the evidence-based evaluation of rat third degree burn model construction and evaluation of alternative indicators, uses the Delphi method to further refine and clarify the model construction items and methods to standardize the rat third-degree burn model construction process and evaluation indicators.

## Materials and methods

### Study design

Delphi is a consulting and decision-making technology summarized and proposed by Rand Corporation in 1964 that can be applied to almost any field. Its core is to conduct several rounds of anonymous correspondence to solicit the opinions of experts. The basic features of Delphi are panel selection, anonymity, iteration, controlled feedback, and statistical aggregation of responses^5,6^. Select experts who have been engaged,and hold deputy senior titles or above, and have rich work experience. consult the opinions of each expert member through correspondence, email, etc. The experts independently complete the questionnaire survey content according to the questionnaire prepared by the research group. The experts do not exchange the survey content with each other, but only contact the researchers . By repeatedly filling out the questionnaire, the opinions of all parties are collected to form a consensus among experts. We evaluated the methods used by the Delphi procedure organizers to send the responses back to the panel. More specifically, we determined whether the experts were informed of both the response of the group and their own individual response (individual feedback) to each item. For each study, we recorded the type of feedback, which was defined as qualitative when a summary of the panel’s comments was sent to each participant and quantitative when simple statistical summaries illustrating the collective opinion (e.g., central tendency and variance) were sent to each participant.

The prediction and evaluation expert group will summarize and sort out the opinions of each round and send them to each expert as reference materials for analysis and judgment and new argumentation opinions. After being repeated many times, the opinions gradually tend to be consistent, and a relatively consistent and reliable conclusion or scheme is obtained. Its essence is to use the collective knowledge and experience of experts to obtain the measurement conclusion by selecting a group of experts to fill in the consultation form many times. Because the Delphi method has the characteristics of anonymity, feedback and statistics, and the statistics, analysis and feedback of expert opinions in the investigation process give full play to the role of information feedback and information control, the Delphi method has been widely used in various fields, not only in the field of prediction, but also in the establishment of various evaluation index systems and the determination process of specific indicators^7,8^. We used a Delphi method in this study, the boundary value method to determine the index of third-degree burn model of rats.

### Building the preliminary indicator pool

We designed a systematic review before the Delphi survey to collect evaluation indicators and descriptions. The literature search was implemented in the MEDLINE (Ovid), EMbase (Ovid), BIOSIS Previews (Ovid), CBM, CNKI, VIP, WanFang Data databases to collect initial tertiary indicators. Some articles were tentatively ruled out for not being relevant to the theme according to the keywords and abstracts, and 15 articles were considered. After evaluation of team members, a burn model of rats with 10 primary indicators and 33 secondary indicators was preliminarily formed. The primary indicators were ① depth of burn, ② methods of induction, ③ burn sites, ④ induction temperature, ⑤ induction time, ⑥ anesthesia, ⑦ skin preparation, ⑧ housing post surgery, ⑨ intervention post surgery, and ⑩ assessment criteria (Table 1).

**Table 1:** The summary of the findings for the various parameters of the models.

Modeling parameters	Subgroup	*n*	%
Depth of burn	I°	3	7.89
	II°	19	50.00
	III°	8	21.05
	I–II°	1	2.63
	II–III°	6	15.79
	Death	1	2.63
Methods of induction	High temperature liquid	6	40.00
	High temperature solid	6	40.00
	High temperature steam	1	6.67
	Mixed fuel	1	6.67
	Infrared radiation	1	6.67
Burn sites	Back	14	93.33
	Abdomen	1	6.67
Induction temperature	45–65 °C	1	6.67
	80–100 °C	10	66.67
	> 200 °C	2	13.33
	Not reported	2	13.33
Induction time	< 1 min	13	86.67
	1–10 min	1	6.67
	> 20miin	1	6.67
Anesthesia	Pentobarbital sodium	6	40.00
	Chloral hydrate	2	13.33
	Ketamine and xylazine	2	13.33
	Uratan	1	6.67
	Ketamine	1	6.67
	Not reported	1	6.67
Skin preparation	Chemical method	4	26.67
	Physical method	5	33.33
	Not reported	6	40.00
Housing post surgery	Reported	3	20.00
	Not reported	12	80.00
Intervention post surgery	Intervention	3	20.00
	No intervention	12	80.00
Assessment criteria	Macroscopic assessment	15	100.00
	Microcosmic assessment	15	100.00

### Using the Delphi method to build a third-degree burn model of rat

The Delphi expert survey included three parts: basic information of a group of experts, assessment of their familiarity with model building indicators evaluation, and evaluation of the constructed index system, along with comments. The first and second stages of the Delphi method encompass a process through which 15 expert’s complete anonymous questionnaire. This research plan selects 15 experts, including 7 clinical medical experts, 8 basic research experts and evidence-based methodology experts from different hospital in China. To solicit input from a diverse group, panelists were recruited using purposive sampling. And they do not know each other, and there is one-to-one communication between the investigator and every expert. The familiarity of indicators and the judgment of indicators was important and scored on a scale from 1 to 5 by these experts. These scores were based on theoretical analysis, work experience, understanding of domestic and foreign peers, and perception.

We conducted two rounds of the Delphi survey and data analysis, and the results were carried out by 15 experts via email or questionnaire survey. Explains the purpose, significance, content, requirements and precautions of the study in detail, and invites experts to evaluate and provide opinions on various indicators.

## Data analysis

### Expert positive coefficient

The positive coefficient of experts is expressed by the questionnaire recovery rate, reflecting the active input of experts; a recovery rate greater than 70% is considered good^9^. A total of 15 copies were distributed in the first round of the Delphi expert survey in this study, and 15 copies were recovered, with an effective recovery rate of 100%. Delphi experts distributed 15 copies in the second round of investigation and recovered 13 copies. The effective recovery rate was 86.67%, indicating that the positive coefficient of the two rounds of experts was good.

### Expert authority coefficient

The expert authority coefficient (Cr) is the arithmetic mean of the judgment coefficient (Ca) and familiarity coefficient (Cs), namely, Cr = (Ca + Cs)/2, where Cr ≥ 0.7 indicates acceptable reliability. Ca represents the evidence at the time of expert judgment, and Cs represents the expert’s familiarity with the problem^10^. Ca is calculated based on expert judgment and the degree of influence of each index. In this research, experts used terms such as “practical experience”, “theoretical analysis”, “understanding of peers” and “insight” as judgments. The evaluation criteria^11^ are shown in Table 2. Then, the evaluation criteria are added to the Ca of each indicator. When Ca = 1, the judgment has a greater influence on the expert; when Ca = 0.5, the influence on the expert’s judgment is moderate; when Ca = 0, it has no obvious influence on the expert’s judgment^12^. Cs refers to the expert’s familiarity with the problem. This study used a scale method to calculate the expert’s familiarity with the problem from 0 to 1 (1 = very familiar, 0.8 = familiar, 0.6 = relatively familiar, 0.4 = generally familiar, 0.2 = not very familiar, 0 = unfamiliar) and calculated the average statistical familiarity of the consultant.

**Table 2:** Judgement basis and the degree of influence.

Judgment scores	Judgment criterion			
	Practical experience	Theoretical analysis	Understanding of peers	Insight
0–1	0.3	0.1	0.1	0.1
2–3	0.4	0.2	0.1	0.1
4–5	0.5	0.3	0.1	0.1

### Concentration of expert opinions

The concentration of experts' opinions is described by $M_{j}$ and *Kj*, where $M_{j} = \frac{1}{{m_{j} }}\sum\limits_{i = 1}^{m} {C_{ij} }$, in the formula, means the average scores of indicator *j* by experts, means the number of experts participating in indicator *j* evaluation, and means the specific score value of expert *i* on the importance of indicator *j*.

The full score frequency is described by *Kj* , $k_{j} = \frac{{m\prime_{j} }}{{m_{j} }}$ . In the formula, *m*_*j*_ represents the number of experts who participated in indicator evaluation *j*, and *mj′* represents the number of experts who gave full marks. The value of *Kj* is between 0 and 1, and *Kj* can be used as a supplementary indicator of $M_{j}$. The larger *Kj* is, the greater the proportion of experts who give full marks to the indicator, and the more important the indicator is. $Vj = \frac{\delta j}{{Xj}}$ where *Vj* represents the coefficient of variation of *j* indicators; $\delta j$ represents the standard deviation of the *j* indicator; *Xj* represents the mean of the *j* indicator. The smaller the *Vj* is, the higher convergence of opinions. In this study, the boundary value was used to screen the evaluation indexes, and the full score frequency, arithmetic mean and coefficient of variation were calculated according to the importance score of each index. The calculation method of the boundary value of full score frequency and arithmetic mean was “boundary value = mean standard -standard deviation”, and those whose score was higher than the boundary value were selected. The calculation method of the variation coefficient boundary value is as follows: “boundary value = mean + standard deviation”, and those whose score is lower than the boundary value are selected. In the above three measurement, the indicators that do not meet the requirements of the three scales will be eliminated. Indicators with one or two scales that do not meet the requirements should be selected after discussion. At the same time, the opinions of experts are fully considered.

Expert coordination is an important indicator for judging the consistency of indicators between experts, including Kendall's W coordination coefficient and the coefficient of variation of each indicator. The coefficient of variation is an important basis for indicator deletion^13^. Kendall’s W coordination coefficient test was used to judge the degree of expert coordination, and *P* < 0.05 was considered statistically significant. The larger Kendall’s W coefficient is, the higher the degree of expert coordination and the higher the consistency of expert opinions. When Kendall's W value > 0.4, the research coordination is better, and when Kendall's W value > 0.5, consulting work can be completed. In this study, Kendall's W test was performed on the expert coordination coefficient of each indicator.

### Statistical analysis

The databases were established using Excel 2016. SPSS 20.0 statistical software was used to calculate the positive coefficient, authority and coordination coefficient of participants to prove the effectiveness of the two rounds of Delphi expert consultation. According to the second round of expert feedback, we calculated the effectiveness of the Delphi process, and based on discussions with experts, used the boundary value method to modify or delete the unqualified indicators. Finally, we can form the final model indicator system according to the indicators that are calculated based on the results of the questionnaire. *P* < 0.05 was considered statistically significant.

## Ethics approval

Because this is only a study on the method of animal model construction and does not involve specific animal experiments, this study protocol was granted a waiver of ethics approval by the West China Hospital Research Ethics Board, and informed consent was waived, all methods were performed in accordance with the Declaration of Helsinki.

## Results

### Basic information of experts

Selecting experts according to our research characteristics requires both academic authoritative experts in this field and experts from the front line. Because our research is a rat burn model, we selected clinical medical experts in the field of burn and plastic surgery and animal experimental experts. In terms of working years and professional titles, we also considered this and selected experts with different working years and professional titles. A total of 15 experts were selected for this consultation study, including 7 clinical medical experts (46.67%) and 8 animal research experts (53.33%). There were 5 experts (33.33%) with less than 15 years of work experience, 8 experts (53.33%) with 15–30 years of work experience and 2 experts (13.33%) with more than 30 years of work experience (Table 3).

**Table 3:** Characteristics of the expert panel.

Characteristic	Round1, *n* (%)	Round2, *n* (%)
**Professional field**		
Clinical medicine	7 (46.67)	5 (38.46)
Animal research	8 (53.33)	8 (61.54)
**Experience**		
Less than 15 years	5 (33.33)	5 (38.46)
Between 15 and 30 years	8 (53.33)	6 (46.15)
More than 30 years	2 (13.33)	2 (15.38)
**Title**		
Associate professor	10 (66.67)	9 (69.23)
Professor	5 (33.33)	4 (30.77)

### Authority coefficient and coordination coefficient

In the two-phase Delphi process, the expert authority coefficient values were 0.73 and 0.67. In the first round, Kendall's W value was 0.435. We carried out the second round of expert consultation on the basis of the first round, and the Kendall's W value was 0.530, so the consultation work was ended.

### Delphi process

Based on the model construction and evaluation alternative indicators obtained from the previous evidence-based evaluation, the Delphi method was used to further refine and clarify the model construction items and methods. Eight primary indicators, 16 secondary indicators and 54 tertiary indicators were preliminarily formed. In this study, self-evaluation is used to judge the degree of expert authority. In the first round of indicators, there are 29 indicators with a degree of expert authority above 0.75, 22 indicators between 0.5 and 0.75 and 3 indicators below 0.5. In the second round, there were 8 indicators with an authority level of experts above 0.75, 36 indicators were between 0.5 and 0.75, and 2 indicators were lower than 0.5. It can be seen that the authority level of indicators was high. In the first round of expert consultation, 15 experts evaluated the results of the indicators (Table 4). The experts put forward 11 opinions for the first round of consultation according to the indicators (Table 5). According to the results of the first round of expert consultation and the opinions of the experts, the boundary value method was used to analyze and modify the alternative evaluation indicators (Supplemental Table 1). A total of 11 indicators were deleted, including 2 secondary indicators: local anesthesia and analgesia. Nine tertiary indicators were deleted, including more than 200 °C, ketamine, lidocaine, bupivacaine, vaccination, analgin, lappaconitine, dexmedetomidine and model construction time consumption. Add a new indicator which was ionizing radiation. There was one indicator updated, and the wound was pale. After referring to expert opinions, a third-degree burn model of rats with 8 primary indicators, 13 secondary indicators and 46 tertiary indicators was finally formed (Table 6). Through the analysis of the boundary value of each indicator (Supplemental Table 2), when constructing a third-degree burn model of rat, it is suggested to use the electric scald instrument less than 80 °C as the burn induction method on the back of the rats. Barium sulfide was used for skin preparation, sodium pentobarbital was used for anesthesia, and attention was given to the postoperative feeding environment of the rats. Ringer's lactate solution with less influence was recommended for postoperative intervention. The macroscopic and microscopic observation results of the burn model, the success rate of model construction, rat mortality, the incidence of complications and the resource consumption of model construction were evaluated.

**Table 4:** The first round of index system based on Delphi method.

Primary indicators	Secondary indicators	Tertiary indicators	$M_{j}$	$\delta j$	*Vj*	*Kj *(%)	Cs	Ca	Cr
Methods of induction	High temperature solid	Electric scald instrument	7.80	1.80	0.23	7.80	0.790	0.873	0.832
		Hydrothermal flask	5.00	2.40	0.48	5.00	0.450	0.787	0.619
		Water bath hot steel bar	5.73	2.05	0.36	5.73	0.590	0.827	0.709
	High temperature liquid	Hot-water bath	7.33	1.62	0.22	7.33	0.770	0.900	0.835
		Water spray injury cup	5.13	2.36	0.46	5.13	0.490	0.780	0.635
		Water bath high temperature gauze	5.27	2.14	0.41	5.27	0.550	0.780	0.665
	Contact combustion	Skin application fuel	4.67	2.67	0.57	4.67	0.510	0.813	0.662
	Thermal radiation	Infrared heater	4.67	2.82	0.60	4.67	0.470	0.780	0.625
Burn sites	Back		9.07	1.34	0.15	9.07	0.920	0.927	0.924
	Abdomen		5.00	3.18	0.64	5.00	0.520	0.813	0.667
	Buttock		3.93	3.04	0.77	3.93	0.400	0.747	0.574
Induction temperature	Less than 80 °C		4.93	2.86	0.58	4.93	0.640	0.853	0.747
	Between 80 and 100 °C		8.40	1.25	0.15	8.40	0.770	0.940	0.856
	More than 200 °C		3.40	3.44	1.01	3.40	0.320	0.753	0.537
Anesthesia	General anesthesia	Pentobarbital sodium	7.67	2.89	0.38	7.67	0.850	0.913	0.882
		Chloral hydrate	6.20	2.76	0.45	6.20	0.670	0.840	0.755
		Ketamine	4.13	2.99	0.72	4.13	0.360	0.760	0.560
		Isoflurane	5.73	3.43	0.60	5.73	0.440	0.813	0.627
		Serazine	2.87	2.03	0.71	2.87	0.230	0.713	0.472
		Uratan	3.73	2.86	0.77	3.73	0.320	0.753	0.537
		Diethyl ether	5.40	3.09	0.57	5.40	0.510	0.847	0.679
	Local anesthesia	Lidocaine	3.87	3.56	0.92	3.87	0.450	0.767	0.609
		Bupivacaine	3.27	2.89	0.88	3.27	0.270	0.707	0.489
Skin preparation	Chemical method	Barium sulfide	4.67	2.98	0.64	4.67	0.390	0.787	0.589
		Sodium sulfide	6.80	3.23	0.47	6.80	0.750	0.873	0.812
	Physical method	Razor	4.73	3.84	0.81	4.73	0.490	0.780	0.635
		Push shear	6.80	3.19	0.47	6.80	0.710	0.867	0.789
Housing post surgery	Rearing environment	Rearing temperature	8.13	2.78	0.34	8.13	0.810	0.920	0.865
		Rearing humidity	8.07	2.79	0.35	8.07	0.800	0.920	0.860
		Environmental ventilation	7.87	2.78	0.35	7.87	0.790	0.900	0.845
		Ambient light	7.20	3.06	0.42	7.20	0.730	0.907	0.819
		Rearing density	8.07	2.74	0.34	8.07	0.800	0.913	0.857
		Selection of bedding material	7.53	3.12	0.41	7.53	0.730	0.907	0.819
	Rearing food	Feed	6.73	3.45	0.51	6.73	0.730	0.900	0.815
		Drinking water	7.13	3.32	0.47	7.13	0.770	0.907	0.839
Intervention post surgery	Prevention of shock	Lactate Ringer's solution	6.87	3.40	0.50	6.87	0.710	0.880	0.795
		Hyperoxia compound sodium chloride	4.53	3.54	0.78	4.53	0.350	0.740	0.545
		Disinfection of animal living environment	7.07	3.47	0.49	7.07	0.750	0.880	0.815
	Prevention of infection	Vaccination	2.07	2.35	1.14	2.07	0.240	0.680	0.460
		Penicillin, generation I cephalosporin	5.00	3.69	0.74	5.00	0.600	0.807	0.704
		Analgin	3.40	3.28	0.97	3.40	0.440	0.727	0.584
	Analgesia	Lappaconitine	2.73	2.86	1.05	2.73	0.320	0.720	0.520
		Dexmedetomidine	2.80	2.97	1.06	2.80	0.310	0.707	0.509
Assessment criteria	Macroscopic results	Wound color	8.73	1.53	0.17	8.73	0.880	0.940	0.910
		Blister formation	7.67	2.57	0.34	7.67	0.800	0.900	0.850
		Eschar formation	8.53	1.63	0.19	8.53	0.880	0.927	0.904
	Microscopic results	Epidermis injury	7.53	3.05	0.41	7.53	0.870	0.940	0.905
		Dermal injury	8.13	2.31	0.28	8.13	0.840	0.927	0.884
		Subcutaneous injury	8.67	1.74	0.20	8.67	0.870	0.927	0.899
	Other comments	Success rate of model construction	8.47	1.54	0.18	8.47	0.830	0.927	0.879
		Model construction time consumption	7.33	2.36	0.32	7.33	0.840	0.920	0.880
		Model building resource consumption	6.87	2.70	0.39	6.87	0.760	0.880	0.820
		Mortality of rats	8.07	1.95	0.24	8.07	0.790	0.907	0.849
		Incidence of complications	7.93	2.24	0.28	7.93	0.750	0.893	0.822

$M_{j}$, means; $\delta j$, standard deviation; *Vj*, coefficient of variation; *Kj*, full score frequency; Ca, judgment coefficient; Cs, familiarity coefficient; Cr, expert authority coefficient.

**Table 5:** Summary of expert opinions after the first round.

Primary indicators	Expert opinions
Methods of induction	① Increase of ionizing radiation; ② Water scald is better to master, and the consistency of animals is better, but the time of 3rd degree burns should be explored, and it may not reach 3rd degree burns; ③ the two methods of water bath scald and solid thermal scald are easier to control and standardize, and have high repeatability; ④ Adding more accurate positioning equipment such as laser thermal burn equipment should be considered
Burn sites	None
Induction temperature	None
Anesthesia	None
Skin preparation	%1Veet Hair Removal Cream Normal Skin; ② The hair of the rat model with 3rd degree burns can be shaved with an electric razor, and there is no need for chemical drugs to completely remove the hair
Housing post surgery	① Normal SPF animal house environment (barrier system), temperature: 20 ~ 25 °C, relative humidity: 40–70%, ventilation rate: 10–15 times/hour, lighting time: 12 h/12 h alternate lighting every day, feed: SPF Special, drinking water: disinfection. It is best to carry out only one experiment in each house to avoid cross-infection; ② The maintenance of wounds after injury are most relevant to temperature/humidity and stocking density, and is not closely related to the feed and drinking water
Intervention post surgery	① Try not to intervene during the experiment, so as not to cause other factors to interfere with the results and cause misjudgment. The success of the experiment is best guaranteed by environmental control
Assessment criteria	① Increase pathological section observation; ② Model construction resource consumption and model construction time consumption, the former includes the latter, and it is recommended to refine the concept of the former; ③ It is recommended to increase the parameters of scalding pressure and scalded area. Added wound healing assay and eschar removal time assay

SPF, specific pathogen-free.

**Table 6:** The second round of index system based on Delphi method.

Primary indicators	Secondary indicators	Tertiary indicators	$M_{j}$	$\delta j$	*Vj*	*Kj * (%)	Cs	Ca	Cr
Methods of induction	High temperature solid	Electric scald instrument	8.08	1.00	0.12	7.69	0.720	0.753	0.737
		Hydrothermal flask	6.38	1.86	0.29	7.69	0.600	0.720	0.660
		Water bath hot steel bar	5.77	1.89	0.33	7.69	0.560	0.693	0.627
	High temperature liquid	Hot-water bath	7.38	1.44	0.20	7.69	0.680	0.760	0.720
		Water spray injury cup	5.38	1.69	0.31	0	0.480	0.653	0.567
		Water bath high temperature gauze	5.54	1.87	0.34	7.69	0.600	0.707	0.654
	Contact combustion	Skin application fuel	5.15	1.99	0.39	7.69	0.493	0.667	0.580
	Thermal radiation	Infrared heater	4.85	2.41	0.50	0	0.453	0.680	0.567
		Ionizing radiation	4.15	2.54	0.61	7.69	0.400	0.660	0.530
Burn sites	Back		9.15	0.77	0.08	38.46	0.800	0.767	0.784
	Abdomen		5.08	2.92	0.57	15.38	0.533	0.640	0.587
	Buttock		5.92	2.59	0.44	15.38	0.5467	0.660	0.603
Induction temperature	Less than 80 °C		8.62	0.84	0.10	15.38	0.707	0.773	0.740
	Between 80 and 100 °C		4.85	2.32	0.48	0	0.573	0.667	0.620
Anesthesia	General anesthesia	Pentobarbital sodium	8	2.63	0.33	23.08	0.733	0.733	0.733
		Chloral hydrate	7.38	2.02	0.27	23.08	0.733	0.793	0.763
		Isoflurane	5.23	2.97	0.57	0	0.387	0.640	0.514
		Diethyl ether	5.46	2.44	0.45	0	0.533	0.667	0.600
		Uratan	4.23	2.52	0.59	0	0.360	0.633	0.497
		Serazine	3.08	1.98	0.64	0	0.320	0.593	0.457
Skin preparation	Chemical method	Barium sulfide	7.69	2.05	0.27	38.46	0.667	0.74	0.704
		Sodium sulfide	6.08	2.53	0.42	7.69	0.427	0.687	0.557
	Physical method	Razor	7.08	2.56	0.36	23.08	0.653	0.713	0.683
		Push shear	6.15	2.57	0.42	15.38	0.573	0.700	0.637
Housing post surgery	Rearing environment	Rearing temperature	8.08	2.53	0.31	30.77	0.653	0.727	0.690
		Rearing humidity	8	2.48	0.31	30.77	0.707	0.72	0.714
		Environmental ventilation	7.85	2.48	0.32	30.77	0.680	0.707	0.694
		Rearing density	7.46	2.62	0.35	23.08	0.680	0.720	0.700
		Selection of bedding material	7.85	2.44	0.31	23.08	0.627	0.713	0.670
		Ambient light	7.54	2.44	0.32	23.08	0.613	0.693	0.653
	Rearing food	Feed	7.46	2.93	0.39	30.77	0.667	0.720	0.694
		Drinking water	7.46	2.93	0.39	30.77	0.667	0.713	0.690
Intervention post surgery	Prevention of shock	Lactate Ringer's solution	7.62	2.56	0.34	23.08	0.667	0.693	0.680
		Hyperoxia compound sodium chloride	5.85	2.35	0.40	0	0.533	0.647	0.590
	Prevention of infection	Disinfection of animal living environment	7.08	2.70	0.38	23.08	0.640	0.687	0.664
		Penicillin, generation I cephalosporin	4.62	2.50	0.54	0	0.547	0.633	0.590
Assessment criteria	Macroscopic results (visual observation)	Wound was pale	8.85	1.10	0.12	38.46	0.747	0.780	0.764
		Eschar formation	8.77	0.89	0.10	23.08	0.760	0.773	0.767
		Blister formation	7.46	2.71	0.36	23.08	0.693	0.760	0.727
	Microscopic results (pathological section observation)	Subcutaneous injury	9.15	0.77	0.08	28.46	0.760	0.787	0.774
		Dermal injury	8.85	0.95	0.11	23.08	0.760	0.773	0.767
		Epidermis injury	8.85	0.86	0.10	23.08	0.773	0.787	0.780
	Other comments	Success rate of model construction	8.69	0.82	0.09	15.38	0.733	0.773	0.753
		Incidence of complications	8.23	1.37	0.17	15.38	0.720	0.747	0.734
		Mortality of rats	8.31	1.07	0.13	15.38	0.733	0.753	0.743
		Model building resource consumption	7.08	2.13	0.30	15.38	0.653	0.740	0.697

$M_{j}$, means; $\delta j$, standard deviation; *Vj*, coefficient of variation; *Kj*, full score frequency; Ca, judgment coefficient; Cs, familiarity coefficient; Cr, expert authority coefficient.

## Discussion

Animal experiments are the basis of promoting human experiments, and an animal model of disease is a prerequisite to ensure the authenticity and stability of animal experiments. Therefore, the construction of animal models of disease is the most basic and important link in the whole biomedical research chain. In the Web of Science Core Collection database, “search filter of animal studies”^14,15^ and "burn" were used for subject retrieval. Research on burn related animal experiments has been increasing. Approximately 53% of the research was published from 2008 to 2018, and 28% of the studies were published in the past five years. This suggests that burn animal experiments have attracted increasing attention and have become an important part of exploring burn treatment. However, a systematic review of heparin in the treatment of burns has shown that the quality of experimental evidence of heparin in the treatment of burns is not high, which is mainly manifested in inconsistent experimental modeling. In the process of modeling, there are mainly the following problems in burn animal experiments; ① early studies did not adopt the random grouping method, which may lead to selective bias; ② the risk of bias of some subjective evaluation indexes may increase if the blind method is not set; ③ the sample size is not estimated, and some of the experimental samples are small, so the results may not be able to accurately infer the overall parameters; ④ most of the domestic studies lack the statement of animal ethics; and ⑤ no potential conflict of interest was reported. At present, there is no research report on the construction method and standard evaluation system of burn animal models at home and abroad, and most studies only focus on the systematic review of a certain animal model^16–20^.

In this study, the Delphi expert consultation method was used to further refine and clarify the items and methods of model construction based on the establishment of a rat third-degree burn model and the evaluation of alternative indicators. After two rounds of Delphi expert consultation, 8 primary indicators, 14 secondary indicators and 46 tertiary indicators were finally included, according to the importance of the indicators. Experts' opinions were described by average scores, so the importance of each index was ranked according to the average scores.

In terms of methods of induction, the top three interventions are electric scald instruments, hot-water baths and hydrothermal flasks. This may be mainly because these three kinds of equipment have better temperature control, which can provide a more stable and constant scald temperature and will not affect the formation of wounds due to temperature fluctuations. In terms of burn sites, the back is considered the most suitable part. The main reason is that the area of skin on the back is larger than that on the buttock. In addition, the skin on the back is thicker than that on the abdomen. The sensitivity of rats to trauma is lower than that on the abdomen. Therefore, the interference factors of the experiment are reduced, which is conducive to the development of the experiment. In terms of burn induction temperature, experts believe that a lower temperature is more conducive to the control of wound formation; on the other hand, the tolerance of rats is also relatively better to further reduce the experimental interference factors. In terms of anesthesia and skin preparation, pentobarbital sodium and barium sulfide were considered to be a more appropriate intervention. In terms of housing postsurgery, combined with expert opinions, it is suggested to adopt a normal SPF animal room environment (barrier system). The temperature is 20–25 °C, the relative humidity is 40–70%, the ventilation frequency is 10–15 times per hour, the lighting time is 12 h every day, the feed is SPF special, and the drinking water is disinfected. It is best to conduct only one experiment in each room to avoid cross infection. In terms of intervention postsurgery, experts believe that the use of Lactated Ringer's solution is better in reducing postoperative shock to reduce the confounding factors of the experiment and reduce the impact on the experimental results as much as possible. In terms of model evaluation, the evaluation of experimental results should be combined with microscopic and macroscopic results, and the success rate of model construction, mortality of rats, incidence of complications and resource consumption of model construction should be described.

Therefore, it is of great significance to explore the construction methods and evaluation criteria of animal models of diseases to ensure and promote the strict implementation of animal experiments. The standardized animal model construction method and evaluation system can help to ensure the internal authenticity and stability of animal experiments, help peer researchers carry out in-depth research on the basis of the same baseline, and promote the smooth and safe progress of preclinical research to clinical research. Therefore, it is particularly important to use scientific and rigorous methods to build animal burn models to improve the quality and repeatability of research.

However, in the practical application of the Delphi method, experts have confirmatory bias, that is, once experts form wrong opinions or hypotheses at the beginning, in the subsequent decision-making process, they often only pay attention to obtaining the information that supports their original opinions or hypotheses and ignore other information so that the wrong opinions, expectations or hypotheses continue, and the research methods need to be further studied. In addition, the model obtained in this study needs to be verified by further experiments.

## Conclusions

Our study demonstrated that when constructing the third-degree burn model of rats, the electric scald apparatus should be used to carry out the experiment on the backs of rats at less than 80 °C. Barium sulfide should be used for skin preparation. Pentobarbital sodium should be used for anesthesia. At the same time, attention should be given to the postoperative feeding environment of rats. Lactate Ringer's solution should be used for postoperative intervention. The success rate of model construction, mortality of rats, incidence of complications and model building resource consumption were evaluated.

## Supplementary Information

Supplementary Information 1.

Supplementary Information 2.

## Data Availability

The dataset will be available upon request unless there are legal or ethical reasons for not doing so.
