# Supplementary material for: Developing a third-degree burn model of rats using the Delphi method
Source: Sci Rep. 2022 Sep 2;12:13852. doi: 10.1038/s41598-022-18092-0 (PMC9440023; doi:10.1038/s41598-022-18092-0)
Supplement: Supplementary file 1 — Supplementary Information 1. [file 41598_2022_18092_MOESM1_ESM.docx]

Supplemental table 1: the boundary values table of the first round

| indicator | means | coefficient of variation | boundary values |
| --- | --- | --- | --- |
| full score frequency | 22.46 | 19.89 | 2.58 |
| arithmetic mean | 6.10 | 1.90 | 4.20 |
| coefficient of variation | 0.52 | 0.26 | 0.77 |

Supplemental table 2: the boundary values table of the second round

| indicator | means | coefficient of variation | boundary values |
| --- | --- | --- | --- |
| full score frequency | 14.06 | 11.62 | 2.44 |
| arithmetic mean | 5.88 | 1.54 | 4.35 |
| coefficient of variation | 0.28 | 0.15 | 0.43 |
