# Supplementary material for: Developing a third-degree burn model of rats using the Delphi method
Source: Sci Rep. 2022 Sep 2;12:13852. doi: 10.1038/s41598-022-18092-0 (PMC9440023; doi:10.1038/s41598-022-18092-0)
Supplement: Supplementary file 2 — Supplementary Information 2. [file 41598_2022_18092_MOESM2_ESM.docx]

Supplemental table 3: questionnaire for Delphi of third-degree burn model of rats

| Constructing and evaluating alternative metrics of third-degree burn model of rats | | | Suitability | Familiarity | Judgments | | | |
| --- | --- | --- | --- | --- | --- | --- | --- | --- |
|  |  |  |  |  | Experience | Theoretical analysis | Domestic and foreign information | Intuition |
| Primary indicators | Secondary indicators | Tertiary indicators | （0-10） | （0-5） | （0-5） | （0-5） | （0-5） | （0-5） |
| Methods of induction | High temperature solid | Electric scald instrument |  |  |  |  |  |  |
|  |  | Hydrothermal flask |  |  |  |  |  |  |
|  |  | Water bath hot steel bar |  |  |  |  |  |  |
|  | High temperature liquid | Hot-water bath |  |  |  |  |  |  |
|  |  | Water spray injury cup |  |  |  |  |  |  |
|  |  | Water bath high temperature gauze |  |  |  |  |  |  |
|  | Contact combustion | Skin application fuel |  |  |  |  |  |  |
|  | Thermal radiation | Infrared heater |  |  |  |  |  |  |
| Burn sites | Back | None |  |  |  |  |  |  |
|  | Abdomen | None |  |  |  |  |  |  |
|  | Buttock | None |  |  |  |  |  |  |
| Induction temperature | Less than 80℃ | None |  |  |  |  |  |  |
|  | Betweent 80 and 100℃ | None |  |  |  |  |  |  |
|  | More than 200℃ | None |  |  |  |  |  |  |
| Anesthesia | General anesthesia | Pentobarbital sodium |  |  |  |  |  |  |
|  |  | Chloral hydrate |  |  |  |  |  |  |
|  |  | Ketamine |  |  |  |  |  |  |
|  |  | Isoflurane |  |  |  |  |  |  |
|  |  | Serazine |  |  |  |  |  |  |
|  |  | Uratan |  |  |  |  |  |  |
|  |  | Diethyl ether |  |  |  |  |  |  |
|  | Local anesthesia | Lidocaine |  |  |  |  |  |  |
|  |  | Bupivacaine |  |  |  |  |  |  |
| Skin preparation | Chemical method | Barium sulfide |  |  |  |  |  |  |
|  |  | Sodium sulfide |  |  |  |  |  |  |
|  | Physical method | Razor |  |  |  |  |  |  |
|  |  | Push shear |  |  |  |  |  |  |
| Housing post surgery | Rearing environment | Rearing temperature |  |  |  |  |  |  |
|  |  | Rearing humidity |  |  |  |  |  |  |
|  |  | Environmental ventilation |  |  |  |  |  |  |
|  |  | Ambient light |  |  |  |  |  |  |
|  |  | Rearing density |  |  |  |  |  |  |
|  |  | Selection of bedding material |  |  |  |  |  |  |
|  | Rearing food | Feed |  |  |  |  |  |  |
|  |  | Drinking water |  |  |  |  |  |  |
| Intervention post surgery | Prevention of shock | Lactate Ringer's solution |  |  |  |  |  |  |
|  |  | Hyperoxia compound sodium chloride |  |  |  |  |  |  |
|  |  | Disinfection of animal living environment |  |  |  |  |  |  |
|  | Prevention of infection | Vaccination |  |  |  |  |  |  |
|  |  | Penicillin, generation I cephalosporin |  |  |  |  |  |  |
|  |  | Analgin |  |  |  |  |  |  |
|  | Analgesia | Lappaconitine |  |  |  |  |  |  |
|  |  | Dexmedetomidine |  |  |  |  |  |  |
| Assessment criteria | Macro results | Wound color |  |  |  |  |  |  |
|  |  | Blister formation |  |  |  |  |  |  |
|  |  | Eschar formation |  |  |  |  |  |  |
|  | Microscopic results | Epidermis injury |  |  |  |  |  |  |
|  |  | Dermal injury |  |  |  |  |  |  |
|  |  | Subcutaneous injury |  |  |  |  |  |  |
|  | Other comments | Success rate of model construction |  |  |  |  |  |  |
|  |  | Model construction time consumption |  |  |  |  |  |  |
|  |  | Model building resource consumption |  |  |  |  |  |  |
|  |  | Mortality of rats |  |  |  |  |  |  |
|  |  | Incidence of complications |  |  |  |  |  |  |
